# Management of a parenteral opioid shortage using ASHP guidelines

**DOI:** 10.1093/ajhp/zxaa425

**Published:** 2021-01-20

**Authors:** Peter Vo, Daniel A Sylvia, Loay Milibari, John Ryan Stackhouse, Paul Szumita, Megan Rocchio, Michael Cotugno, Caryn Belisle, Charles Morris, Eric Goralnick, Joshua C Vacanti, Lina Matta, Tom Cooley, Angela Triggs, Jon Silverman, John Fanikos

**Affiliations:** 1 Pharmacy Department, Brigham and Women’s Hospital, Boston, MA, USA; 7 Pharmacy Department, Brigham and Women’s Hospital, Boston, MA; 9 Department of Medicine, Brigham and Women’s Hospital, Boston, MA, USA; 10 Department of Emergency Medicine, Brigham and Women’s Hospital, Boston, MA, USA; 11 Department of Anesthesiology, Perioperative and Pain Medicine, Brigham and Women’s Hospital, Boston, MA, USA

**Keywords:** drug shortage, injectable opioids, opioid infusions, patient-controlled analgesia

## Abstract

**Purpose:**

Management of an acute shortage of parenteral opioid products at a large hospital through prescribing interventions and other guideline-recommended actions is described.

**Summary:**

In early 2018, many hospitals were faced with a shortage of parenteral opioids that was predicted to last an entire year. The American Society of Health-System Pharmacists (ASHP) has published guidelines on managing drug product shortages. This article describes the application of these guidelines to manage the parenteral opioid shortage and the impact on opioid dispensing that occurred in 2018. Our approach paralleled that recommended in the ASHP guidelines. Daily dispensing reports generated from automated dispensing cabinets and from the electronic health record were used to capture dispenses of opioid medications. Opioid prescribing and utilization data were converted to morphine milligram equivalents (MME) to allow clinical leaders and hospital administrators to quickly evaluate opioid inventories and consumption. Action steps included utilization of substitute opioid therapies and conversion of opioid patient-controlled analgesia (PCA) and opioid infusions to intravenous bolus dose administration. Parenteral opioid supplies were successfully rationed so that surgical and elective procedures were not canceled or delayed. During the shortage, opioid dispensing decreased in the inpatient care areas from approximately 2.0 million MME to 1.4 million MME and in the operating rooms from 0.56 MME to 0.29 million MME. The combination of electronic health record alerts, increased utilization of intravenous acetaminophen and liposomal bupivacaine, and pharmacist interventions resulted in a 67% decline in PCA use and a 65% decline in opioid infusions.

**Conclusion:**

A multidisciplinary response is necessary for effective management of drug shortages through implementation of strategies and practices for notifying clinicians of shortages and identifying optimal alternative therapies.

**Key Points**

- The ASHP guidelines on managing drug product shortages provide an effective structure for facilitating optimal drug supplies during the time of a significant opioid shortage.
- A pharmacy leadership plays an essential role in drug shortage management through communications with suppliers, administrators, healthcare providers, and patients.
- At a hospital in Massachusetts faced with a shortage of parenteral opioid products, supplies of those medication were rationed through patient prioritization and other strategies so that surgical and elective procedures were not canceled or delayed.

In early February 2018, hospitals first recognized a shortage of parenteral opioids that was expected to last until the end of 2018.^1-4^ The American Society of Health-System Pharmacists (ASHP) published results of a 2018 hospital survey indicating a shortage of parenteral opioid formulations.^5^ In the survey, 68% of respondents indicated that parenteral opioid supplies were limited enough to affect their daily operations and patient care. Morphine, fentanyl, and hydromorphone injectable formulations were most impacted. This shortage was believed to be created by multiple factors. In response to the opioid epidemic in the United States, the Drug Enforcement Administration restricted the amounts of active pharmaceutical ingredients available to pharmaceutical manufacturers. From 2016 through 2018, there was a 52% reduction in opioid production. In 2017, a major manufacturer stopped opioid production, and as it held a large market share, the disruption of this service line directly impacted patient care.^1-6^

In the spring of 2018, our hospital, Brigham and Women’s Hospital in Boston, MA, which is a 793-bed facility within the Mass General Brigham integrated health network, experienced an acute shortage of injectable opioids. In this report, our objective is to describe the application of ASHP Guidelines on Managing Drug Product Shortages during a 1-year period (calendar year 2018).^7^ Our second objective is to describe the changes in opioid dispensing that occurred over the same period.

## Response to the opioid shortage

Recognizing that the opioid shortage would impact patient care, the pharmacy staff communicated the problem to the hospital leadership, who activated the hospital’s emergency incident command system (HEICS) (Table 1). The command system established supply allocation, with prioritization of parental opioid supply to the operating rooms (ORs) and procedural areas, critical intensive care units, and the emergency department (Table 2). Due to experiences with prior shortages, the pharmacy adopted the ASHP guidelines^7^ through an institutional infrastructure. The hospital’s designated drug product shortage team convened every morning to discuss the inventory of opioids that were available and how the supply would be rationed to meet the demands of the patients. The hospital’s resource allocation committee was the source of data on opioid inventories and utilization and served as the working group for generating electronic health record alerts to influence physician prescribing practices. Clinical pharmacists identified substitute opioid therapies, which were vetted through the pharmacy and therapeutics committee on an ad hoc basis, establishing a process for approving alternative therapies.

**Table 1.** ASHP Guideline Recommendations and Action Steps Taken at the Hospital

ASHP Guideline Recommendation	Hospital Action Steps	Participants	Outcome
Hospital emergency incident command system	HEICS activated to communicate opioid shortage and potential impact on patient care. HEICS assisted in rapidly establishing emergency management planning, response, and recovery capabilities for the drug shortage event. HEICS meetings held daily for 3 days and then transitioned to as-needed meetings.	Meetings included Chief Medical Officer, Chief Nursing Officer (both served as Incident Commanders), Chief Communication Officer, Chief at Staff, Director of Emergency Preparedness, Safety Officer, Chief Nursing Officer, Chief Oncology Nurse, Assistant Chief Nursing Officer, Clinical Director of Emergency Department, Medical Director of Peri-Op Services, Associate Chief Nurse of Critical Care, and Associate Chief Nursing of Periprocedural Departments, Director of Neurosurgical Critical Care, Vice Chair of Surgery, Senior Vice President of Ambulatory, Chief Information Officer, Director of Materials Management, Chief of Surgery, Chief Operating Officer, Chief of Fetal Medicine, and Chief Financial Officer.	Call for action and structure established to allow communication from service Chiefs (Medicine, Surgical, ICU, Emergency Department, Peri-Op) downward through the organization.
Drug product shortage team	Team met daily to review reports on parenteral opioid on-hand inventories, review prior day’s utilization, project upcoming day’s deliveries, reviewed surgical cases, and reviewed interventions and substitutions. Team discussed how choices impacted daily demands of surgery, emergency care, and other treatments.	Meetings directed by Assistant Chief Medical Officer and Director of Emergency Preparedness. Meetings included pharmacy managers, nurse managers, surgeons, anesthesiologists, critical care physicians, and Chief Information Officer and Director of Finance.	Working group established for real-time planning, decision-making, and issuing directives for managing the opioid shortage. Priority care areas established. Interventions and substitutions approved and daily plan communicated to clinical staff.
Resource allocation committee	Committee met daily to determine the opioid inventories and agents available; analyzed and reported opioid utilization patterns from patient care areas; established schedule for in-hospital pharmacy compounding; identified opioids needing intervention and generated options substitution; created or adjusted infusion device drug libraries; crafted alerts in electronic health record; and provided advanced notice to affected clinicians.	Meetings orchestrated by Pharmacy Director. Meetings included inpatient pharmacy managers, nurse managers, pharmacy purchasing agents, information system analyst, care coordination, and Assistant Chief Medical Officer.	Working group established for data collection and transformation into reports. Working group created and maintained hospital intranet website for communication, current state, new updates, and dose conversions. Assistant Chief Medical Officer communicated weekly and as needed to clinical staff, division chiefs, public relations, research community, and ethics committee.
Process for approving alternative therapies	Clinical pharmacist pain specialist identified substitutes and provided opioid dosing conversion tables. Anesthesiologists representing the operating room and ambulatory procedural areas (catheterization lab, imaging, endoscopy, etc) collaborated on content.	P&T committee and members (clinical pharmacists, nurses, and physicians) provided review, approval, and support for therapeutic substitutes, policy, and procedure changes.	Opioid dosing conversion tables created and maintained. Drug administration guidelines updated for prescribers and nurses. Clinical pharmacist reported interventions and outcomes to Medical Staff Executive, ICU, and Clinical Improvement Committees.
Process for addressing ethical considerations	Office of ethics representative was available 24/7.	Office of ethics representatives	Communications were not needed for clinician or patient consultation.

Abbreviations: ASHP, American Society of Health-System Pharmacists; HEICS, hospital emergency incident command system; ICU, intensive care unit.

**Table 2.** Operational and Therapeutic Assessment Process and Procedures Used at the Hospital During the Drug Shortage

ASHP Guideline Recommendation	Hospital Action Steps	Participants	Outcomes
Details and duration of shortage	Met with local distributor, drug manufacturer, and group purchasing organization representatives	Pharmacy purchasing agent, pharmacists, manufacturers, local distributors, and group purchasing organization	Staff informed that shortage was predicted to last for 12-18 months Longevity of shortage communicated to clinicians and leaders Short- and long-term planning adjusted
Purchasing from outside pharmacies and in-house compounding or manufacturing	Expanded use of outside vendors to include registered 503B outsourcing facilities Started preparing single-dose syringes from multiple dose vials Purchased active pharmaceutical ingredients (eg, fentanyl, hydromorphone) and established a process for sterile production	Inpatient pharmacy staff	Able to increase hospital’s supply of opioids and developed a process for sterile production of active pharmaceutical ingredients
Inventory on hand	Pharmacy purchasing agents kept on-hand opioid inventory values, including agents, estimated utilization, projected days’ supply on hand, velocity of dispensing, and automated dispensing activity for daily accountability Individual agents tallied, totaled, and reported in MME	Pharmacy purchasing agents, pharmacists, inpatient pharmacy technicians, and service contact designees	Inpatient pharmacy was able to communicate to service contact lines about which agents had a limited supply or which agents to be switched to Rapid determination of the hospital’s ability to schedule and perform procedures
Process for approving alternative therapies and changes in process	Routine substitution of opioids at equianalgesic doses Reduced reliance on patient-controlled and continuous infusion opioid administration Routine ordering limited to acute pain service and critical care attending physicians	Clinical pharmacists, nurses, physicians (critical care, hospitalist, emergency medicine, anesthesia) and P&T committee	Clinical pharmacists served as approvers for all PCA and continuous-infusion opioid use
Therapeutic alternatives	Evaluated therapeutic alternatives to opioids; reviewed formulary restrictions and opioid sparing options Removed restriction for intravenous acetaminophen and liposomal bupivacaine expanding access for both medical and surgical patients	P&T committee, pharmacists, physicians, and nurses	Unlimited supplies available Global increase in use of restricted agents
Patient prioritization	Prioritized operating room procedures, patients on circulatory support, and ICU patients	Hospital emergency incident command system and inpatient pharmacy	Was able to appropriately manage limited formulations of opioids to critical patients
Financial ramifications	Incremental spending reported on monthly financial reports	Pharmacy managers, internal audit, accountants, and financial officers	Dramatic increase in drug spend
Disclosure to patients	Did not have any discussions with patients and families about the drug shortages; did not have any medication safety reports that affected patient care due to the opioid shortage.	Medication safety office and patient family relations staff	None identified
Communication to media	Hospital public relations department communicated to local media, and information provided to local news outlets and news station	Hospital public relations department	Media notification of shortages; media highlighted potential impact of shortages on patient care
Stockpiling restraint	There was no ability to stockpile or engage in speculative purchasing during the shortage	Pharmacy purchasing agents	Future consideration of creating an emergency or disaster inventory

Abbreviations: ASHP, American Society of Health-System Pharmacists; ICU, intensive care unit; MME, milligram morphine equivalents; PCA, patient-controlled analgesia; P&T, pharmacy and therapeutics.

Our pharmacy purchasing staff recognized the onset of the opioid shortage in February 2018. As the month progressed, available parenteral opioid options dwindled from unit dose formulations to multidose formulations, and more products went on back-order status until they were finally were out of stock. Our local distributors confirmed that there was a stuttering pattern of product availability. They communicated that future opioid purchasing would be allocated on the basis of historical purchases. Later in the same month, a manufacturer representative cited plant problems and confirmed that the company’s formulations would not be routinely available until the end of the year.^6^

Available opioids were purchased from wherever supply was available, including alternate local distributors and registered 503B compounding facilities. Both commercial and compounded products that had been previously purchased were now prepared in the pharmacy cleanroom. We scheduled pharmacists, pharmacy technicians, and pharmacy interns to work during off-hours to meet the increased sterile compounding demand.

We used daily dispensing reports generated from automated dispensing cabinets (ADCs) and from our electronic health record for opioid medications on Schedules II through V. We calculated morphine milligram equivalents (MME) dispensed, using the Centers for Disease Control and Prevention tool and publications in the medical literature on formulation conversions, to measure opioid utilization.^8-10^ The MME totals allowed the hospital’s clinical and administrative leaders to quickly determine ability to schedule and perform surgical procedures. For our reporting, we considered opioid formulations dispensed rather than the actual dose administered to patients as a reflection of utilization. We did not capture dispensing of opioid agonist/antagonist analgesics (buprenorphine, butorphanol, nalbuphine, and pentazocine), benzodiazepines, muscle relaxants, hypnotics, or stimulants. Opioids used for non–pain management indications (cough syrups, suppositories, and topical agents) were also excluded. However, we did monitor use of selected non-narcotic analgesics, including oral and intravenous acetaminophen, oral ibuprofen (parenteral ibuprofen is not on the hospital’s formulary), parenteral ketorolac, and liposomal bupivacaine.

Orders for continuous opioid infusions and patient-controlled analgesia (PCA) were carefully reviewed, as they require multiple doses in a delivery reservoir and often generate waste. Whenever possible, PCA and infusions were converted to intravenous opioid bolus dose administration. In addition, a daily goal was to limit opioid infusions to ventilated patients, patients requiring neuromuscular blockers, and those with severe pain who required continuous opioid infusions as a final option.

Soft- and hard-stop clinical decision support alerts were created through the electronic health record to influence prescribing of opioid infusions and PCA medications. (A soft stop allows a clinician to continue to progress past the alert and order an opioid, whereas a hard stop halts opioid prescribing.)

Postoperative pain, acute pain, and chronic pain management patients were transitioned from parenteral to enteral therapy as soon as appropriate. Unit-based clinical pharmacists were equipped with daily patient lists and asked to work with nurses to identify good candidates for de-escalation of therapy from parenteral to oral opioids.

There was daily shifting of parenteral opioid products in various formulations and concentrations to meet expected utilization demands. While available opioid inventories impacted these shifts, short beyond-use dating of pharmacy-compounded opioid medications contributed as well.

During the shortage, nonopioid oral formulations were encouraged and some formulary restrictions were removed. For example, restrictions on intravenous acetaminophen, which limited its use to enhanced recovery after surgery (ERAS) pathways for up to 3 dose administrations in the 24-hour postoperative period, were removed during the last week of March 2018. Intravenous acetaminophen was also supplied to ORs and was utilized in all hospitalized inpatients. Similarly, restrictions limiting use of liposomal bupivacaine to open abdominal ERAS pathways were removed. Liposomal bupivacaine became readily available to physicians in surgical settings as an alternative analgesic agent.

## Results of response to the drug shortage

The ASHP guidelines provided a structure for organizing human resources into teams that facilitated decision making, action steps, and communications for managing the opioid shortage. Parenteral opioid supplies were successfully rationed so that surgical and elective procedures were not canceled or delayed.

During the shortage, opioid dispensing decreased in the inpatient care areas from approximately 2 million MME (in January 2018) to a nadir of 1.4 million MME (in April 2018) before increasing to 2.1 million MME (in October 2018) (Appendix A). Similarly, opioid dispensing decreased in the ORs from 0.56 MME (in January 2018) to a nadir of 0.29 million MME (in June 2018) before increasing to 0.56 MME (in October 2018). These reductions were largely driven by utilization of parenteral hydromorphone, which declined significantly from 0.97 million MME (in February 2018) to 0.16 million MME (in March 2018) before increasing gradually to preshortage levels in the fall (October 2018). Parenteral morphine was substituted for hydromorphone in February and March but then became in short supply. This resulted in utilization declining in April 2018 and remaining at that level throughout the year. For inpatient care, oral opioids and intravenous fentanyl dispensing remained stable throughout the year (Appendix B). In our ORs, fentanyl dispensing declined 58%, from 0.12 million MME (in February 2018) to a nadir of 0.04 million MME (in April 2018) (Appendix C). Similarly, remifentanil dispensing declined by 43%, from 0.39 million MME (in January 2018) to 0.23 million MME (in April 2018). These shortages were offset by sufentanil dispensing, which tripled in March 2018 and remained elevated through the remainder of the year.

The combination of electronic health record alerts and pharmacist intervention produced a 67% decline in PCA use, which was decreased from 1.1 million MME (in February 2018) to 0.36 million MME (in March 2018). Thereafter, PCA use increased steadily until reaching preshortage levels in October 2018 (Appendix D). Likewise, opioid infusions decreased by 65%, from 0.78 million MME (in February 2018) to a nadir of 0.27 million MME (in March 2018) and returned to routine dispensing levels in December 2018.

While there were no significant changes in the dispensing of oral acetaminophen, oral ibuprofen, and parenteral ketorolac each month, dispensing of intravenous acetaminophen and liposomal bupivacaine changed dramatically (Appendix E). Intravenous acetaminophen dispensing rose steadily over several months, reaching a peak total of 4,414 g in November 2018. Liposomal bupivacaine dispensing increased to a peak total of 60.2 g in July 2018.

The 2018 opioid shortage occurred acutely at our institution. Our greatest challenge was managing an approximately 28% reduction in opioid MME dispensing from April through June. When we analyzed data for the entire year, there were decreases in total MME dispensing of 12.2% and 13.6% from dispensing levels in 2016 and 2017, respectively.

## Discussion

Drug shortages have been commonly reported, affecting broad categories of medications and populations of patients.^5-9^ ASHP has published guidelines to help pharmacy departments organize, establish structure, and create actionable steps to manage medication shortages.^7^ We applied these guidelines to manage a parenteral opioid shortage in 2018. The guidelines helped create an overall hospital goal, drive decision making on opioid and nonopioid medication selection, and facilitate communication of substitutions and dose equivalents to clinicians. Furthermore, the guidelines helped allocate pharmacist resources to intercept and change prescribing of PCA and opioid infusions and helped foster transition to therapeutic alternatives. Finally, the guidelines helped sustain this structure and corresponding action steps for the 12 months while the shortage lasted. We found that despite a significant reduction in opioid MME dispensing early in the shortage, no procedures were canceled or delayed. However, there was a dramatic increase in use of nonopioid alternatives, specifically intravenous acetaminophen and liposomal bupivacaine, as the shortage progressed.

Shortages have been attributed to manufacturers, regulatory agencies, and misaligned profits and rewards.^11-14^ Along with rising stress and frustration among providers, pricing of available and alternative medications have risen as well. Researchers at a children’s hospital evaluated the impact of an intravenous opioid shortage on postoperative pain control in a pediatric cardiac intensive care unit.^15^ Despite a switch to available alternative parenteral opioids, the researchers did not find an increase in the rate of uncontrolled pain or an increase in adverse events. These outcomes were attributed, in part, to the education provided by clinical pharmacists. Another study evaluated the rates of prescribing errors in a pediatric intensive care unit before and after a national shortage of fentanyl and injectable benzodiazepines and found no significant differences.^16^ A recent study evaluated the impact of the parenteral opioid shortage on prescribing patterns and pain control in hospitalized cancer patients receiving palliative care. The investigators found that fewer parenteral opioids were prescribed and fewer patients achieved improved pain scores.^2^

Our study had a number of limitations. We used opioid dispensing activity as a surrogate for opioid administrations. We did not capture all medications that could have been dispensed for acute and chronic pain. We did not account for specific opioid doses for patients and for opioids that were wasted or returned to the pharmacy or an ADC. We did not assess pain scores or patient satisfaction. Finally, we did not take into consideration patient acuity, number of surgeries, or patient admission volume.

The study also had some strengths. Numerous publications alert clinicians of existing drug shortages, but few provide recommendations on steps to take in managing a drug shortage. As our hospital provides care for a broad scope of medical and surgical patients, the experience described here is likely applicable to many US hospitals.

The management of an opioid shortage starts with early identification of the problem, ensuring an ample supply of alternative analgesic agents, and reservation of opioids for patient cases that cannot do without them. These strategies can be implemented regardless of any type of drug shortage to limit the severity and duration of insufficient supply.

## Conclusion

ASHP guidelines on managing drug shortages provided a structure for creating teams and action steps to coordinate a hospital response. These guidelines outlined strategies for our pharmacy department to take a leadership role in communicating with clinicians, administrators, and patients about the opioid shortage. Also, the guidelines specified an operational and therapeutic assessment of the shortage in order to implement temporary procedures, alternative therapeutics, information system changes, and patient prioritization. Our use of these guidelines was essential to our success in mitigating the impact of the shortages on the quality of patient care.

## Figures and Tables

**Appendix A:**
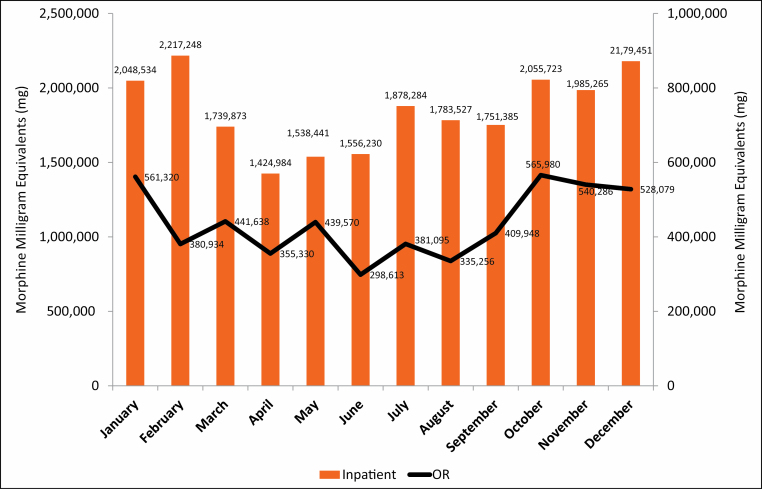
Opioid dispensing for inpatient and operating room (OR) care in 2018

**Appendix B:**
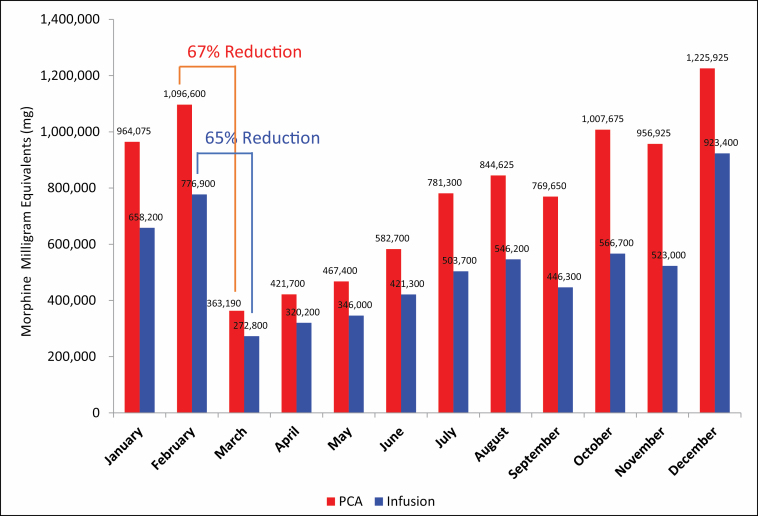
Opioid patient-controlled analgesia (PCA) and opioid infusions dispensed for inpatient and operating room care in 2018

**Appendix C:**
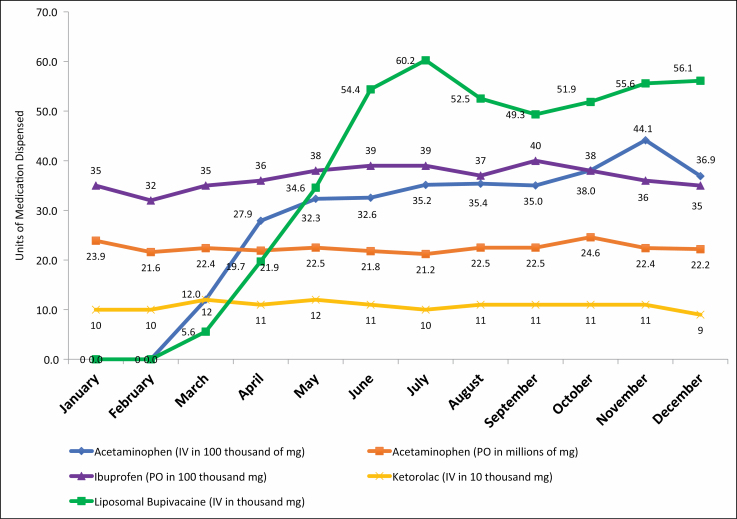
Adjunctive nonopioid medications dispensed to operating rooms and inpatients in 2018

**Appendix D:**
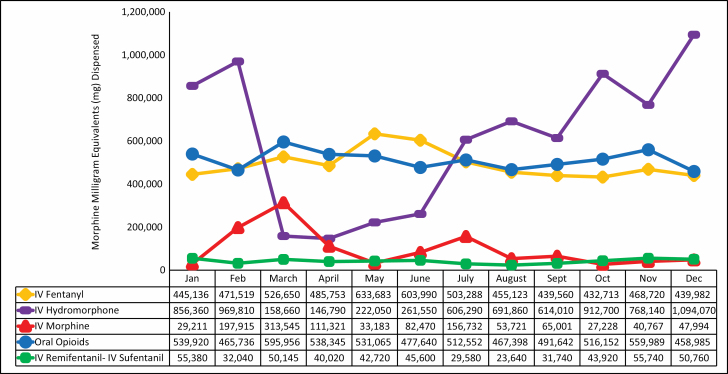
Opioids dispensed to hospitalized inpatients in 2018

**Appendix E:**
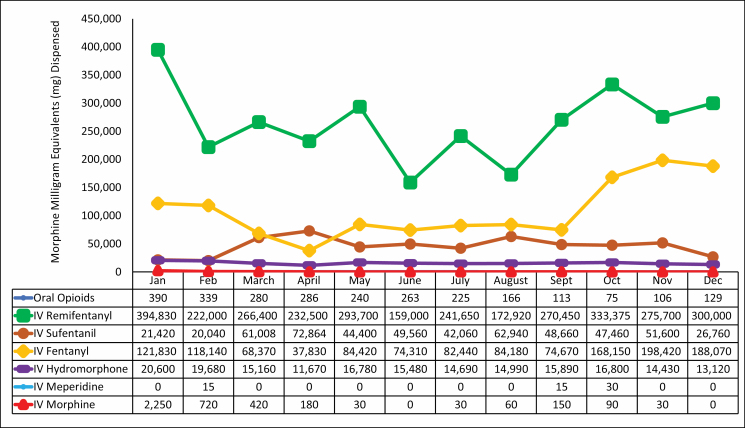
Opioid medications dispensed to operating rooms (ORs) in 2018
